# Assessing High-Intensity Acceleration Efforts Using Local Positioning System—Introducing the Concept of the Relative Acceleration Threshold to Ice Hockey

**DOI:** 10.3390/sports14020062

**Published:** 2026-02-04

**Authors:** Christian Bielmann, Karin Fischer-Sonderegger, Quirin Söhnlein, Wolfgang Taube, Markus Tschopp

**Affiliations:** 1 Department of Elite Sport, Swiss Federal Institute of Sport Magglingen SFISM, 2532 Magglingen, Switzerland; 2 Department of Neurosciences and Movement Science, University of Fribourg, 1700 Fribourg, Switzerland; 3 Lausanne Hockey Club, 1000 Lausanne, Switzerland

**Keywords:** ice hockey, external load, acceleration, local positioning system, player tracking, wearable technology, team sports, kinematic

## Abstract

Current methods for assessing acceleration efforts (acc_efforts_) in ice hockey do not account for the influence of initial skating speed on maximal voluntary acceleration capacity, which may lead to a biased evaluation of acc_effort_ intensity. In this study, we introduce the conceptual approach of the relative acceleration threshold (rel_threshold_) to ice hockey and outline its potential benefits for the assessment of acc_efforts_. Locomotion data derived from observations of 17 players across 10 official games were used to model the initial-skating-speed-dependent maximal voluntary acceleration capacity (a_max_–v_init capacity_), from which a team-specific rel_threshold_ was determined (rel_threshold_75%_ = 3.23 − 0.365v_init_), and, subsequently, applied to assess acc_efforts_ alongside a fix threshold set at 2 m·s^−2^ (fix_threshold_2_). Differences in acc_efforts_ depended on the method used (rel_threshold_75%_ vs. fix_threshold_2_) as well as the playing position when using the rel_threshold_75%_. The fix_threshold_2_ reported 89.1 ± 35.8% more acc_efforts_ than the rel_threshold_75%_. However, only one-third of these acc_efforts_ exceeded rel_threshold_75%_, which is considered indicative of neuromuscularly intense acc_efforts_ according to the modeled a_max_–v_init capacity_. Moreover, at skating speeds above 4 m·s^−1^, the fix_threshold_2_ only assessed a negligible number of acc_efforts_, whereas the rel_threshold_75%_ assessed 27.2 ± 9.3% of all its acc_efforts_. In line with established theoretical rationales, the observational findings of this study suggest that an acceleration threshold adapted to the initial skating speed offers a conceptually more valid approach to assessing acc_efforts_ in ice hockey.

## 1. Introduction

Local positioning systems (LPSs) enable the objective quantification of players’ on-ice external kinematic loads using various indicators. LPSs have recently gained popularity in applied research and training monitoring in ice hockey due to their increased availability and affordability [1,2,3]. However, selecting the most appropriate and pertinent sport-specific indicators to meaningfully describe activity profiles is challenging [2,4,5,6]. In this context, to capture the frequently occurring metabolically and mechanically demanding acceleration activities, which are not well represented by the use of distance- and speed-based measures alone, an increased focus on tracking-system-derived acceleration measures has been highlighted in team sports in general [7,8,9]. Within tracking-system-derived acceleration measures, acceleration activities are generally operationalized using different approaches, such as the distance covered or time spent in predefined acceleration zones, the averaging of instantaneous acceleration values over time, or the number of discrete acceleration efforts performed above a set acceleration threshold, with the latter representing the most frequently reported approach in the literature [10,11].

Specifically in ice hockey, where the gliding mode of locomotion allows distances to be covered with reduced physical strain, the importance of quantifying acceleration activities has been suggested to be even more important for comprehensively assessing the external kinematic load [6,12,13,14,15]. Nevertheless, there is currently no consensus on how acceleration activities should be operationally defined. In contemporary ice hockey research using LPSs, acceleration activities have been assessed exclusively by quantifying discrete acceleration efforts (acc_efforts_) using the traditional fixed acceleration threshold method (fix_threshold_ method, often also referred to as absolute threshold) set at 2 m·s^−2^ [2,16,17,18] and, on a single occasion, at 2.41 m·s^−2^ and 2.78 m·s^−2^ [6].

However, the use of a fix_threshold_ to assess acc_efforts_ leads to a systematic methodological bias, as the conclusions of several studies have empirically indicated that the maximal voluntary acceleration capacity progressively decreases with increasing locomotion speed [19,20]. Simply put, the greater the locomotion speed from which an acc_effort_ is initiated, the lower the maximal voluntary acceleration capacity; up to the point when the peak locomotion speed is reached, the acceleration capacity is equal to zero because no more force can be applied [21,22]. To overcome this issue and improve the validity of acc_efforts_ evaluation, Sonderegger et al. [19] proposed the ‘relative acceleration threshold method’ (rel_threshold_ method) by accounting for the influence of initial speed on the maximal voluntary acceleration capacity. This method relies on identifying discrete acc_efforts_ using the acceleration zero-crossings as the start and endpoint of an acc_effort_. As such, in soccer, initial-speed-dependent maximal voluntary acceleration capacities have been modeled first, using isolated linear sprint testing [19,23] and, more recently, using training locomotion [24] and game data [25]. Subsequently, the peak acceleration measured for each discrete acc_effort_ is evaluated against the maximal voluntary acceleration capacity that can be achieved from that particular initial locomotion speed. Any effort exceeding the 75% threshold is classified as an intense acc_effort_ [19].

Accounting for the influence of the initial speed on the maximal voluntary acceleration capacity appears particularly relevant in ice hockey. As an intermittent team sport played on ice with unlimited substitutions, ice hockey has a unique game structure characterized by repetitive brief periods of intense activity, typically lasting 30 to 90 s, followed by passive recovery phases of 3 to 5 min on the bench [26,27,28]. Approximately half of the distance covered in a game is spent skating at speeds exceeding 17 km·h^−1^. This results in average game speeds ranging from 12 to 17 km·h^−1^ [16,26,29,30,31], depending on various factors such as playing position, tactical system, opposing team, competition standard, and the number of power-play (numerical advantage) and penalty-kill (numerical disadvantage) situations. This is notably higher than the average game locomotion speeds observed in other team sports (e.g., soccer: 6.7 km·h^−1^ [4]; rugby league: 5.1 km·h^−1^ [32]). Therefore, ice hockey players may engage in more frequent acc_efforts_ initiated from higher locomotion speeds, accentuating the relevance of contextualizing acc_effort_ magnitudes based on the underlying locomotion speed. This may be particularly relevant for forwards, as previous studies have shown that they cover more distance in high-speed skating zones than defensemen [2,16,30,31], which provides a basis for hypothesizing that they may perform acc_efforts_ more frequently from higher locomotion speeds.

The purpose of this study was to introduce the concept of the relative acceleration threshold to ice hockey. Specifically, rather than aiming to establish a fully validated methodological approach, this study seeks to outline the potential advantages of applying a relative acceleration threshold to assess acc_efforts_ using LPS. Accordingly, the first part of the study aims to determine a team-specific relative acceleration threshold by modeling the initial-skating-speed-dependent maximal voluntary acceleration capacity (a_max_–v_init capacity_) using official game locomotion data. The aims of the second part of the study are (i) to explore differences in the number of acc_efforts_ captured by the traditional fix_threshold_ and the proposed rel_threshold_, and (ii) to investigate positional differences using the rel_threshold_, thus evaluating the approach’s ability to discriminate between positions based on expected differences. Forwards are hypothesized to perform more acc_efforts_ initiated from higher skating speeds than defensemen.

## 2. Materials and Methods

### 2.1. Design and Subjects

This applied research study was observational in design and included pre-existing in-game locomotion data initially collected as part of a regular monitoring routine of a professional team competing in the highest tier of Swiss Ice Hockey (National League) during the 2023–2024 season. Game locomotion data were collected from all rostered field players during ten consecutive regular-season home games played over a period of 48 days. The total sample included 25 players, who were designated as defensemen (*n* = 9; age: 27.5 ± 3.9 years; stature: 185.1 ± 7.6 cm; body mass. 88.2 ± 7.1 kg) or forwards (*n* = 16; age: 27.5 ± 3.8 years; stature: 181.7 ± 5.4 cm; body mass. 85.8 ± 6.3 kg). The inclusion criterion for Part 1 of the study—the threshold determination analyses—was a minimum of 100 cumulative effective playing minutes throughout the observational phase, resulting in *n* = 17 athletes (*n* = 6 defensemen; *n* = 11 forwards). This criterion was applied to ensure that enough acc_efforts_ were captured throughout the whole skating speed spectrum accelerating maximally or close to maximally, which represents an important methodological prerequisite for building robust and valid models. The inclusion criteria for Part 2 of the study were (i) players with match-files with a minimum of 10 min of effective playing time, in order to limit possible biases from low-minute-playing players inflating relative values, and (ii) players who played a minimum of two games throughout the observational phase, resulting in a total of *n* = 22 players (*n* = 8 defensemen; *n* = 14 forwards) with an average of 8.77 ± 2.36 (range: 2–10) games per player. The games were played on an International Ice Hockey Federation (IIHF) European-sized hockey rink (60 m × 30 m; 1800 m^2^). The study was approved by the Institutional Review Board of the Swiss Federal Institute of Sport Magglingen (245_LSP_09_2024) and was conducted in accordance with the Declaration of Helsinki.

### 2.2. Measurement System

Players’ in-game locomotion data were collected using an ultra-wideband local positioning system (LPS) operating at a sampling rate of 20 Hz (Kinexon, Munich, Germany, software version: 11.4.11; firmware version: release/6.11.0-sports-lps). In compliance with the manufacturer’s guidelines, the LPS sensor was tightly secured in a patch on the posterior side of the players’ shoulder pads, near the superior one-third of the scapula, to avoid unnecessary device movement. The manufacturer’s software-derived speed and acceleration data was used for analyses. This data was based on their latest proprietary postprocessing filtering methods at the time of data retrieval (September 2024), which consisted of an unscented Kalman filter and a second-order Butterworth low-pass filter with a cut-off frequency of 1 Hz. Recently, a study confirmed the validity and reliability of the Kinexon LPS [33]: the peak speed and acceleration demonstrated acceptable intersensor reliability (CV < 10%) when comparing two sensors worn by the same player, and they showed near-perfect correlations in instantaneous speed, peak speed, and acceleration (all r’s > 0.892) compared to a previously validated robotic sprint device [33]. Data were trimmed to only include the effective playing time for each player through the synchronization of the LPS with the game clock. Once downloaded, acceleration and speed data traces were subsequently processed using a custom-written MATLAB script (Version R2020a, MathWorks Inc., Natick, MA, USA).

### 2.3. Method for Detecting Players’ Discrete acc_effort_

Players’ discrete acc_efforts_ were detected from the acceleration trace using the zero-crossing method [34]. The start of an acc_effort_ was defined as the moment the acceleration signal turned positive and ended when the signal returned 0 m·s^−2^ (i.e., the velocity stopped increasing). The acc_effort_ duration (a_duration_), speed increase (v_delta_), peak acceleration magnitudes attained during the effort (a_max_) and skating speed from which the acceleration was initiated (v_init_) were retrieved for each effort. In cases where the acceleration trace fell below 0.5 m·s^−2^ for more than five consecutive data points without turning negative, and then subsequently increased again, the velocity preceding the second rise was considered v_init_. This criterion was applied because it has been shown that, during abrupt deceleration phases immediately followed by re-acceleration, the instantaneous acceleration trace may be filtered in such a way that the actual short deceleration phase is obscured, potentially merging two or even several distinct acc_efforts_ misleadingly into a single zero-crossing [35]. Accordingly, this dip-rule was implemented to correct for this potential artifact and to ensure the proper alignment of v_init_ with the corresponding a_max_. Subsequently, for a zero-crossing to finally be considered an acc_effort_, the following criteria had to be satisfied: First, a_duration_ had to last at least 1.15 s, corresponding to approximatively two skating cycles according to Kaartinen et al. [36]. Secondly, the player needed to gain a minimum speed increase of 3.6 km·h^−1^ (v_delta_). Only acc_efforts_ fulfilling the above-mentioned criteria were retained for analysis. Acc_efforts_ that did not meet the criteria were considered either (i) a representation of a negligible mechanical load to the player—negligible when considered in isolation but indeed contributing cumulatively to the player’s overall load, an effect not addressed in the present study—or (ii) signal noise.

### 2.4. Modeling Initial-Skating-Speed-Dependent Maximal Voluntary Acceleration Capacity

**Figure 1:**
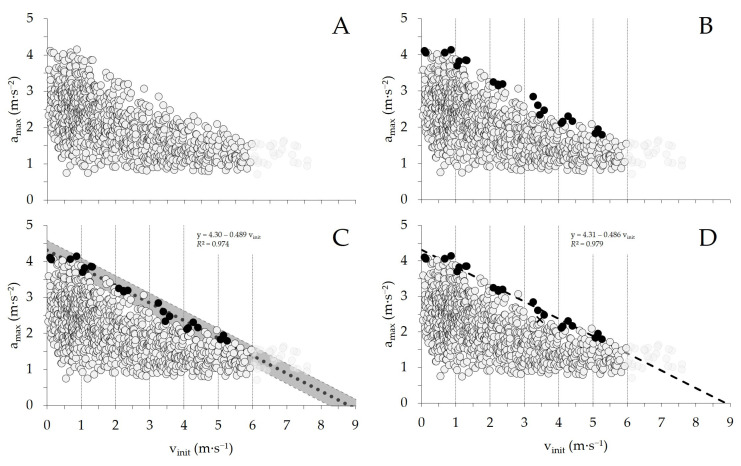
Determination of subjects’ individual initial-skating-speed-dependent maximal voluntary acceleration capacity (a_max_–v_init capacity_) regression line. (**A**) All acceleration efforts (acc_efforts_) plotted with maximal acceleration (a_max_) versus initial skating speed (v_init_). (**B**) For each 1 m·s^−1^ v_init_ subinterval up to 6 m·s^−1^, the four highest acc_efforts_ were selected (black, dots). (**C**) First linear regression with 95% prediction intervals fitted to the selected acc_efforts_. (**D**) Final linear regression after exclusion of outliers outside the 95% prediction interval (black, cross), yielding the subjects’ individual a_max_–v_init capacity_ regression line.

The endpoint of the first part of the study was to determine a team-specific relative acceleration threshold. This threshold was derived from the in-game modeled initial-speed-dependent maximal voluntary acceleration capacity (a_max_–v_init capacity_), which was obtained by linear regression analysis of game locomotion data and was used as an operational marker for the external mechanical limits of the neuromuscular system to accelerate at different initial skating speeds. As such, each subject’s individual a_max_–v_init capacity_ regression line was determined using the following procedure (see Figure 1, A–D for a graphical illustration using one representative player): First, all their acc_efforts_ performed throughout the observation phase were compiled and plotted in a two-dimensional diagram with the a_max_ (the maximal acceleration reached for each effort) on the y-axis and the v_init_ (the speed from which the action was initiated) on the x-axis (Figure 1A). Next, the four acc_efforts_ with the highest acceleration values detected within each 1 m·s^−1^ speed subinterval up to 6 m·s^−1^ (e.g., 0–1, 1–2, 2–3 m·s^−1^) were selected (black dots, Figure 1B). This procedure was limited to v_init_ subintervals up to 6 m·s^−1^ because not all players or positions consistently accelerated from higher initial speeds. Accordingly, data above 6 m·s^−1^ were excluded from the threshold determination procedure. Then, a first simple linear regression was fitted to the selected acc_efforts_ and the 95% prediction intervals were calculated (Figure 1C). Finally, acc_efforts_ outside of the 95% prediction interval were removed and a second and final regression equation, which excluded the outliers, was calculated to improve the overall accuracy of the model (Figure 1D). The resulting regression equation characterizes the subjects’ individually modeled a_max_–v_init capacity_, where the y-intercept describes the theoretical maximal acceleration when starting at rest and the slope indicates the decrease in a_max_ as a function of increasing v_init_.

In contrast to previous studies in soccer, which typically included the selection of the highest available acc_efforts_ to compute a_max_–v_init capacity_ regression lines, we opted for a more conservative a_max_–v_init capacity_ regression line determination. The rationale behind this decision is that the selection of relevant acc_efforts_ from the cloud of points to determine the a_max_–v_init capacity_ regression lines has been shown to be challenging and prone to measurement errors [25]. We therefore argue that a more conservative a_max_–v_init capacity_ regression line determination provides a more robust and less error-prone basis for anchoring the relative acceleration threshold. Two key methodological decisions were made to approximate the true unknown a_max_–v_init capacity_ regression line: First, based on the number of games compiled, we opted for 1 m·s^−1^ speed subintervals. Narrower speed intervals might have resulted in certain intervals not containing acc_efforts_ located at or in close proximity to the true unknown a_max_–v_init capacity_ regression line, i.e., they may have included acc_efforts_ that are too low to represent maximal efforts, thereby inevitably increasing the deviation from the true unknown a_max_–v_init capacity_ regression line. However, broader speed intervals would have reduced the total number of acc_efforts_, which would have made the regression parameters less robust and increased the uncertainty associated with the prediction intervals, ultimately compromising the ability to identify outliers [25]. The second decision concerned the number of acc_efforts_ within the subintervals. Similarly, we reasoned that a higher count of acc_efforts_ would yield more robust regression parameters and reduce the uncertainty associated with the prediction intervals used for outlier detection. However, too many acc_efforts_ within the subinterval could negatively affect regression parameter accuracy, as an increasingly large number of submaximal acc_efforts_ would have been accounted for, thus increasing the deviation from the true unknown a_max_–v_init capacity_ regression line [25]. In an unpublished preliminary study, we compared various configurations and found that four data points per 1 m·s^−1^ speed subinterval resulted in the highest coefficient of determination (R^2^) for this observational timeframe. Therefore, this configuration was adopted for the current study. However, it should be noted that different configurations tested during preliminary internal analysis did not substantially influence the regression parameters (not published).

Subjects’ individual a_max_–v_init capacity_ regression line coefficients (i.e., y-intercept and slope) were then averaged to calculate a team-level grand mean regression line (by adding up the individual regression coefficients and dividing the total by the number of subjects). The relative acceleration threshold (rel_threshold_75%_) was subsequently derived as 75% of the grand mean. A summary of the modeled individual- and team-level a_max_–v_init capacity_ regression lines, coefficient of variation in the regression coefficients (CV%), and the resulting team-specific rel_threshold_75%_, which was further used for Part 2 of the study, are reported at the beginning of the Results section.

Additionally, acc_efforts_ were assessed using the commonly used fix_threshold_-method set at 2 m·s^−2^ (fix_threshold_2_) using the same criteria (i.e., a_duration,_ v_delta_) for an acc_effort_ to be included. Moreover, acc_efforts_ assessed using both computational methods were further categorized into initial-skating-speed bands from which they were initiated using a fixed bandwidth of 2 m·s^−1^ intervals (0–2 m·s^−1^, very low speed; 2–4 m·s^−1^, low speed; 4–6 m·s^−1^, moderate speed; >6 m·s^−1^, fast speed).

### 2.5. Statistical Analyses

Unless otherwise stated, all data are presented as mean ± standard deviation (SD) and were analyzed using SPSS Version 28.0.1.0 for Windows (SPSS Inc, Chicago, IL, USA). The goodness of fit (R^2^) was used to assess the quality of the linear fit of the individually modeled a_max_–v_init capacity_ regression lines. Descriptive statistics were calculated to indicate both the absolute and normalized number of acc_efforts_ assessed during official match play for both acc_effort_ computational methods. The normalized number of acc_efforts_ is expressed relative to the effective playing time and scaled on a per minute basis to account for interindividual differences in playing time. To investigate differences between the two methods (fix_threshold_2_ vs. rel_threshold_75%_) and between playing positions (forwards vs. defensemen), the mean individual number of acc_efforts_ across all matches played during the observation period was used for statistical analyses. Normalized values were used to assess differences, in order to ensure comparability across playing positions while accounting for interindividual differences in effective playing time. The Shapiro–Wilk test revealed that the number of acc_efforts_ assessed using both methods and their resulting differences were normally distributed (*p* > 0.05), allowing parametric tests to be applied. Paired-sample *t*-tests were used to assess differences between the two computational methods (fix_threshold_2_ vs. rel_threshold_75%_) regarding the number of acc_efforts_ captured in total and across the various initial-skating-speed bands. Independent-sample *t*-tests were used to evaluate positional differences in the number of acc_efforts_ performed using the rel_threshold_75%_—again, in total and across the different initial-skating-speed bands. To interpret the meaningfulness of the differences, Cohen effect-size (*d*) statistics are reported, which can be interpreted as follows: <0.2, trivial; 0.2 to 0.6, small; 0.6 to 1.2, moderate; 1.2 to 2.0, large; and >2.0, very large [37]. Statistical significance was set at *p* ≤ 0.05.

## 3. Results

### 3.1. Team-Specific Relative Acceleration Threshold Determination

**Figure 2:**
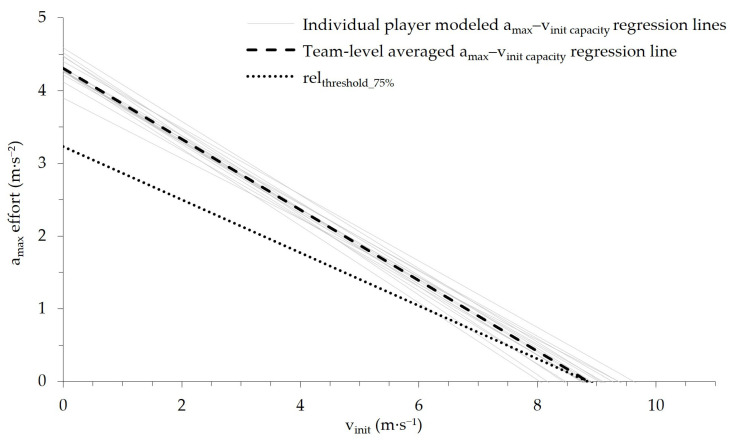
Individually modeled a_max_–v_init capacity_ regression lines of the *n =* 17 ice hockey players (gray solid lines), the corresponding team-level averaged a_max_–v_init capacity_ regression line (black, dashed line), and the resulting team-specific rel_threshold_75%_ (black, dotted line).

Part 1 of this study aimed to determine a team-specific relative acceleration threshold using the averaged, individually modeled a_max_–v_init capacity_ regression lines. The goodness of fit (R^2^) of the individually modeled a_max_–v_init capacity_ regression lines was 0.94 ± 0.02 (range: 0.89–0.98), with a corresponding standard error of the estimate of 0.21 ± 0.04 m·s^−2^. The resulting team-level mean y-intercept (i.e., theoretical maximal acceleration capacity from standing) was 4.31 ± 0.16 m·s^−2^ (95% confidence interval (CI) [4.22, 4.39]), with a coefficient of variation (%CV) of 3.72%, and the mean slope was 0.48 ± 0.03 (95% CI [0.47, 0.50]) with a CV of 6.17%. Figure 2 depicts the individual- and the team-level modeled a_max_–v_init capacity_ regression lines. Applying a margin of 75%, the resulting team-specific relative acceleration threshold (rel_threshold_75%_ in m·s^−2^; v_init_ in m·s^−1^) was determined as follows, with values in parentheses indicating 95% CI:

rel_threshold_75%_ = 3.23 (3.17 to 3.29) − 0.365 (−0.352 to −0.377)v_init_ (1)

### 3.2. Descriptive Match Analysis

The average effective playing time was 16.0 ± 2.7 min per game. When using the rel_threshold_75%_-method, players performed 26.1 ± 6.5 acc_efforts_ per game, of which 50.2 ± 7.7%, 22.7 ± 3.2%, 19.9 ± 5.4%, and 7.2 ± 4.1% were initiated from ‘very low’, ‘low’, ‘moderate’, and ‘fast’ skating speeds, respectively. At the same time, using the fix_threshold_2_ method, the number of acc_efforts_ performed was 47.9 ± 8.2 per game, of which 81.1 ± 3.5%, 17.7 ± 3.2%, 1.2 ± 0.7%, and 0.0% were initiated from ‘very low’, ‘low’, ‘moderate’, and ‘fast’ skating speeds, respectively.

### 3.3. Differences in the Number of Assessed acc_efforts_ Between the fix_threshold_2_ and rel_threshold_75%_ Method

Table 1 presents the mean number of assessed acc_efforts_ per effective playing minute for the two computational methods as well as the paired *t*-test statistics comparing the two methods. In total (i.e., all initial-skating-speed bands combined), the fix_threshold_2_ method assessed, on average, 1.37 ± 0.32 (89.1 ± 35.8%) more acc_efforts_ per minute than the rel_threshold_75%_ method. When analyzed using initial-skating-speed bands, the fix_threshold_2_ method assessed significantly more acc_efforts_ in the two lowest speed bands, with mean differences of 1.63 ± 0.23 and 0.16 ± 0.05 acc_efforts_ per minute in the ‘very low’ and ‘low’ initial-skating-speed bands between the two methods, respectively. Conversely, in the ‘moderate’ and ‘fast’ initial-skating-speed bands, the rel_threshold_75%_ method assessed significantly more acc_efforts_, with mean differences of 0.30 ± 0.11 and 0.13 ± 0.08 acc_efforts_ per minute between the two methods, respectively. Notably, using the fix_threshold_2_ method, acc_efforts_ initiated from a ‘moderate’ skating speed were only rarely detected, while no acc_efforts_ from a ‘fast’ skating speed were observed.

**Figure 3:**
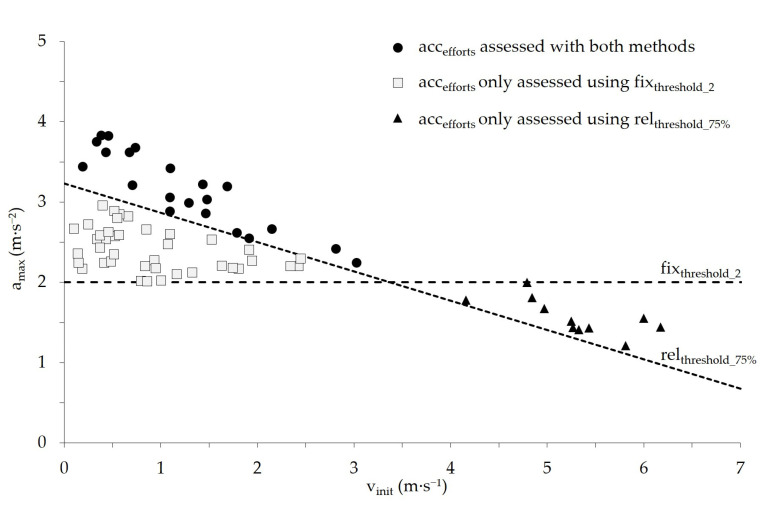
Visualization of acc_efforts_ of one representative player assessed during a single game based on the applied computation methods. The player’s effective playing time in this game was 17:59 min. Acc_efforts_ concurrently assessed using both methods are shown as black circles (*n* = 21), acc_efforts_ only assessed using rel_threshold_75%_ appear as triangles (*n* = 11), and acc_efforts_ assessed only using fix_threshold_2_ appear as squares (*n* = 40).

Figure 3 shows an example from one player during a single game, illustrating all acc_efforts_ identified using both computational methods. Of all the acc_efforts_ shown in this example (Figure 3), 55.6% were detected exclusively using fix_threshold_2_ and located below the 75% margin of the team-level modeled a_max_–v_init capacity_ regression line (Figure 3; squares), whereas 15.3% were detected exclusively using rel_threshold_75%_ and located below fix_threshold_2_ (Figure 3; triangles).

### 3.4. Positional Differences Using rel_threshold_75%_

**Figure 4:**
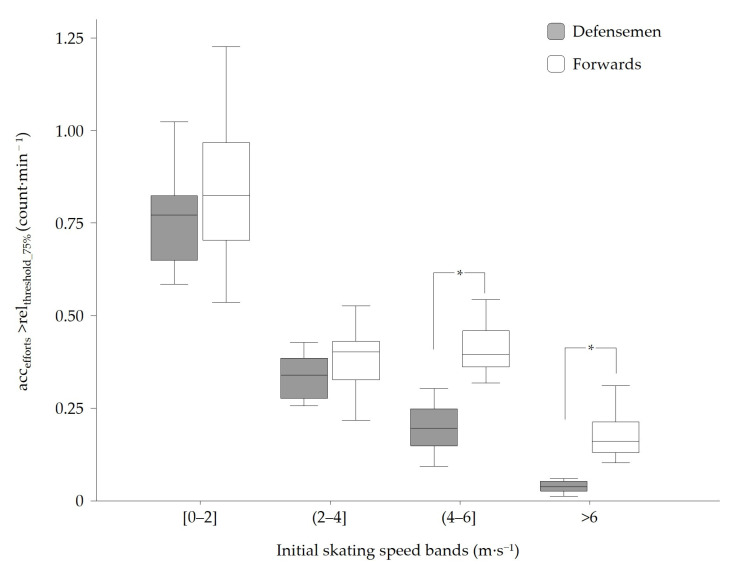
Boxplots showing positional differences between forwards and defensemen in the number of acc_efforts_ above the rel_threshold_75%_. Values are expressed per minute across the four skating speed bands. Boxes indicate the medians and interquartile ranges; whiskers show the spread. * Significant positional difference (*p* < 0.05).

Exploring positional differences using the rel_threshold_75%_ method revealed that, in total (i.e., all initial-skating-speed bands combined), forwards performed significantly more acc_efforts_ per minute played than defensemen (1.83 ± 0.33 vs. 1.34 ± 0.23, respectively, *t*(20) = −3.70, *p* < 0.001, *d* = 1.64). A further analysis within the four initial-skating-speed bands is displayed in Figure 4 and showed no statistically significant positional differences in the ‘very low’ (0–2 m·s^−1^; *t*(20)= −1.05; *p* = 0.307) and ‘low’ (2–4 m·s^−1^; *t*(20) = −1.45, *p* = 0.164) initial-skating-speed bands, but a medium-sized effect (*d* = 0.47 and 0.64, respectively). In the ‘moderate’ (4–6 m·s^−1^) initial-skating-speed band, forwards performed significantly more acc_efforts_ than defensemen (0.42 ± 0.07 vs. 0.20 ± 0.07, respectively; *t*(20) = −6.65; *p* <0.001; *d* = 2.95). In the ‘fast’ (>6 m·s^−1^) initial-skating-speed band, forwards (M = 0.17; SD = 0.05) also performed significantly more acc_efforts_ than defensemen (0.17 ± 0.05 vs. 0.04 ± 0.03, respectively; *t*(20) = −6.17; *p* < 0.001; *d* = 2.74).

## 4. Discussion

Currently, the influence of initial skating speed on maximal voluntary acceleration capacity is not accounted for when assessing acc_efforts_ in ice hockey—which, according to Sonderegger et al. [19] and in accordance with the widely accepted macroscopic inverse linear force–velocity relationship [22,38,39,40], confounds the evaluation of acc_effort_ intensity. Against this background, the aim of the present study was to introduce an established approach from soccer—the relative acceleration threshold—to ice hockey. The gliding nature of ice hockey, as well as the flying and unlimited substitutions, result in a generally faster pace of play compared to other team sports. Thus, accounting for the influence of initial skating speed on maximal voluntary acceleration capacity may be particularly relevant when assessing acc_efforts_ using LPS.

In the first part of the study, the initial-skating-speed-dependent maximal voluntary acceleration capacity (a_max_–v_init capacity_), which is considered indicative of the mechanical limit of the neuromuscular system to accelerate at different locomotion speeds, was modeled for the first time in ice hockey using game locomotion data. At the team level, the a_max_–v_init capacity_ regression line shows that the maximal voluntary acceleration capacity from a standing start reaches a magnitude of 4.31 ± 0.16 m·s^−2^ and progressively decreases by 0.48 ± 0.03 m·s^−2^ for each 1 m·s^−1^ increase in initial skating speed. The present study confirms the hypothesized influence of initial skating speed on maximal voluntary acceleration capacity in the context of ice hockey. From a theoretical perspective, the modeled a_max_–v_init capacity_ suggests that an acceleration magnitude of 2 m·s^−2^ can only be considered maximal when initiated at a skating speed of approximately 16.9 km·h^−1^. When initiated from a stationary start, the same acceleration magnitude corresponds to approximately 46.4% of the maximal voluntary acceleration capacity. This illustrates the importance of applying a relative acceleration threshold, as it contextualizes acceleration magnitudes with respect to the underlying skating speed, which in turn is suggested to enable a more valid assessment of acc_effort_ intensity.

This empirical observation is theoretically grounded within the overarching and well-documented principle of the macroscopic force–velocity relationship which characterizes the external mechanical limits of the neuromuscular system to apply force and, consequently, accelerate at different movement speeds in multi-joint acyclic and cyclic ballistic tasks [38], such as pedaling [41,42], squatting [43], or sprinting [39].

### 4.1. Differences in the Number of Assessed acc_efforts_ Between the fix_threshold_2_ and rel_threshold_75%_

In line with the theoretical rationale, differences were observed between the fix_threshold_2_ and rel_threshold_75%_ in the number of acc_efforts_ assessed. In the present study, the total number of acc_efforts_ assessed using the fix_threshold_2_ was more than 1.8 times that assessed using the rel_threshold_75%_ (47.9 ± 8.2 vs. 26.0 ± 6.5 per game). This difference was particularly evident in acc_efforts_ initiated from ‘very low’ and ‘low’ initial-skating-speeds, where, on average, 2.9 and 1.5 times more acc_efforts_ were assessed, respectively. While only a negligible number of acc_efforts_ initiated from speeds > 4 m·s^−1^ were assessed using the fix_threshold_2_, the rel_threshold_75%_ captured a considerable proportion of 27.8 ± 6.5% of all its acc_efforts_ assessed above this initial speed threshold. These acc_efforts_, which would have been disregarded when applying fix_threshold_2_, occurred at ‘moderate’ and ‘fast’ initial skating speeds and were located in close proximity to the modeled mechanical limits of the neuromuscular system. They may therefore be associated with high neuromuscular demands. The number of acc_efforts_ identified using the fix_threshold_2_ was comparable to the values reported in previous ice hockey studies employing the same threshold [2,16,18]. However, the number of assessed acc_efforts_ using the rel_threshold_75%_ was more closely aligned with findings from qualitative, semi-automated, video-based analyses in modern ice hockey, which may point towards greater ecological validity, although the operational definitions differ fundamentally and this interpretation requires caution. For instance, Brocherie et al. [26] evaluated five games using a structured, qualitative observational grid and reported an average of 14 “*maximal efforts with drastic forward lean*” in elite male international ice hockey. Similarly, but with a slightly different observational grid, Jackson et al. [44] identified an average of 12 “*Forward start*” and 5 “*high/maximal intensity forward skating*” per game in male collegiate-level ice hockey. The discrepancy between the number of acc_efforts_ reported using fix_threshold_2_ and those identified by subjective evaluations could be attributed to the associated bias of the fix_threshold_ method, which is purported to overestimate the intensity of acc_efforts_ initiated from low initial skating speeds, while simultaneously underestimating those from higher speeds. Our data indicate that a considerable proportion of approximately 60% of the acc_efforts_ assessed using the fix_threshold_2_ were below 75% of the team-level modeled a_max_–v_init capacity_. Consequently, these acc_efforts_ were likely not identified by the raters as relevant and/or high-intensity acc_efforts_, which may serve as a possible explanation for the discrepancies in the number of acc_efforts_ between fix_threshold_2_ and subjective evaluations of trained experts.

### 4.2. Positional Differences Using rel_threshold_75%_

In a second step, this study investigated positional differences in the number of acc_efforts_ performed between defensemen and forwards when using the relative acceleration threshold. The aim was to evaluate the method’s ability to discriminate between playing positions based on expected positional differences. Previous studies have shown that forwards cover more distance in high-skating-speed zones (>17 km·h^−1^) [2,16,30,31] and reach higher peak speeds during gameplay [2,16], which provided a basis for hypothesizing that forwards may engage in more frequent acc_efforts_ initiated from higher locomotion speeds compared to defensemen. Our results were consistent with this assumption, as we observed only nonsignificant small-to-moderate positional differences in the two lowest initial-skating-speed bands (*p* = 0.307 and 0.164, respectively), while significant differences with very large effect sizes were identified in the ‘moderate’ and ‘fast’ initial-skating-speed bands. These neuromuscularly intense acc_efforts_ initiated at moderate-to-fast skating speed typically occur during forwards’ specific actions, such as forechecking, breakouts, breakaways, dumps in the offensive zone, and offensive rushes, which represent about 32% of the intense acc_efforts_ among forwards. Therefore, capturing these efforts is essential to more comprehensively assess players’ in-game acceleration demands and, moreover, to avoid overlooking positional differences.

### 4.3. Practical Implications and Limitations of the Descriptive Match Analysis Results

Practitioners using LPSs should consider integrating a relative acceleration threshold to assess acc_efforts_ into their monitoring routines, as it enables a more comprehensive assessment of the acc_efforts_ players are exposed to. As such, this approach supports player health and performance through improved training prescription and load management. Beyond reporting the prevalence of acc_efforts_, this study also characterized the initial speeds from which these efforts were initiated, showing that a substantial proportion (32.2% in forwards and 17.9% in defensemen) were initiated from skating speeds above 14.4 km·h^−1^. This finding has direct practical relevance, as acc_efforts_ initiated from higher initial skating speeds likely result in different skating mechanics, such as altered push-off characteristics, stride frequency, and neuromuscular demands [36,45]; accordingly, practitioners should ensure sufficient exposure to such acc_efforts_ in training and consider incorporating more targeted drills to specifically develop this capacity.

The descriptive match analysis results discussed above are subject to several limitations. As the study used official game locomotion data from a single team’s home games, the reported results may have been influenced by the team’s tactical orientation in combination with opponent strength. Therefore, the findings may not generalize to other teams or competitive contexts. Moreover, averaging per-match acc_efforts_ across multiple games precluded an examination of match-to-match variability, potentially masking meaningful fluctuations between games. However, due to the highly intermittent character of the game, even when considered on a per-match basis, averaged values obscure the most demanding playing phases that players are subjected to (also known as the “worst-case scenario” [46]). Those conducting future studies should focus their attention on the most intense playing phases and their density to elucidate the games’ peak acc_efforts_ demands, which may be more informative and, thus, more adequately prepare athletes for the demands of the game [3] and/or serve as a variable for scientific investigations of the game at a finer level of granularity.

### 4.4. Methodological Challenges in Detecting Players’ Discrete acc_effort_ and Modeling a_max_–v_init capacities_ Using Game Observations

Several methodological decisions related to data processing, discrete acc_effort_ detection, and the modeling of a_max_–v_init capacitiy_ warrant consideration. For instance, to reduce noise in positioning data, which is often amplified during highly dynamic, multidirectional movements in team sports [47], data-cleaning techniques are commonly applied; however, different cleaning approaches can substantially alter the acceleration trace [11,48]. Soft filtering will maintain a high level of noise, whereas strong filtering could eliminate important characteristics of a signal [48]. In the present study, the manufacturer’s processed data was used, as it had recently been validated against a robotic sprint device. Furthermore, it is a simple and efficient way to obtain data, which potentially increases the practical use and replicability of the study results for researchers using the same tracking system. However, different filtering settings could have resulted in different acceleration traces, which would in turn influence the study results.

A second aspect concerns the application of the minimal effort duration (MED) for an acc_effort_ to be considered (also known as the ‘dwell time’ [49]). The MED is typically used to avoid classifying measurement-error-related spikes as true efforts [49,50]. As the MED substantially influences the number and type of detected acc_efforts_ [49,50,51], its definition is critical. In contrast to the common approach of defining the MED as the time above a threshold, we applied it to the duration of the acceleration zero-crossing. The selected MED of 1.15 s was an assumption-based methodological choice, intended to represent approximately two skating cycles according to a recent biomechanical study [36], and may have resulted in the exclusion of shorter, yet potentially meaningful acceleration efforts.

A further aspect that is often overlooked is the method used to identify the end of an effort [49]. As shown by Fischer-Sonderegger et al. [35], abrupt deceleration phases followed by immediate reacceleration can obscure the acceleration zero-crossing when strong filtering is applied. In such cases, implementing a dip-rule may mitigate filter-induced artifacts and support a more robust discrete acc_effort_ identification.

While the detection of discrete acc_efforts_ represents a necessary first step, the subsequent modeling approach for determining a_max_–v_init capacity_ based on these events entails additional methodological options, each associated with specific assumptions and trade-offs. One central aspect relates to the context in which the data were obtained. For practical reasons—specifically the availability of game locomotion data—we determined the a_max_–v_init capacity_ from game locomotion data rather than from dedicated sprint tests, as originally conducted by Sonderegger et al. [19]. In this regard, previous research in team sports suggested that in situ (i.e., game- or training-based) locomotion data offers a viable means to approximate peak neuromuscular capacities when sufficient exposure is considered. For example, Andrey et al. [25] demonstrated that at least 2–3 games are required in soccer to obtain valid in situ estimates of maximal capacities, whereas Maviel et al. [52] reported that approximately two matches are sufficient in rugby to achieve reliable estimates. However, the minimum exposure required to obtain valid and reliable in situ estimates is likely sport-specific and may additionally be influenced by broader contextual factors, including player motivation, opponent strength, and tactical orientation, among other factors. In the present study, a_max_–v_init capacities_ were only considered for players who accumulated at least 100 min of effective playing time—corresponding to approximately six full matches and thus representing a comparatively cautious exposure duration when interpreted relative to minimum exposure requirements reported in other team sports. This minimum exposure criterion was selected to increase the likelihood that the dataset contained a sufficiently broad and dense distribution of maximal acc_efforts_ across the relevant skating speed spectrum. In line with this rationale, players with lower playing time showed more variable model fits (i.e., lower R^2^ values) and larger residual errors, suggesting that insufficient exposure may lead to the incomplete representation of maximal acc_efforts_ and, thus, to less valid estimations of the a_max_–v_init capacity_.

Relatedly, once sufficient exposure has produced a broad cloud of in-game acc_efforts_, the estimation of a_max_–v_init capacity_ requires additional methodological decisions concerning data point selection for model fitting to reduce the risk of bias arising from positive random measurement error or the inclusion of submaximal acc_efforts_ [25]. Accordingly, in the present study, the modeling choices regarding the width of the skating speed subintervals, the number of acceleration efforts retained within each interval, and the exclusion of efforts initiated above 6 m·s^−1^ were made with the intention of approximating the true yet unknown a_max_–v_init capacity_ under in situ conditions. Notably, extrapolating the model beyond the skating speed range used for its derivation is associated with a certain degree of uncertainty.

Taken together, the methodological decisions made in the present study were guided by a combination of previous research in soccer (e.g., [25]) and sport-specific theoretical considerations, and therefore remain assumption-based rather than supported by prior empirical validation in ice hockey. While the concept of the relative acceleration threshold itself is supported by well-established theoretical reasoning and empirical evidence, the validity of the specific methodological approach used in the present study for discrete acc_effort_ detection and the subsequent modeling of a_max_–v_init capacities_ remains to be validated in the context of ice hockey, which was beyond the scope of the present investigation. Future research should therefore seek to determine the most valid methodological approach to discrete acc_effort_ detection and the subsequent modeling of a_max_–v_init capacities_ in ice hockey, ideally supported by validation against a gold-standard motion capture system or cross-referenced with synchronized video footage to qualitatively evaluate the accuracy of different methodological operationalizations.

Against this background, combined with the use of a convenience sample comprising 17 professional male ice hockey players from a single team, the relative acceleration threshold (Equation (1)) determined in the present study should be interpreted with caution. Its generalizability to other populations or contexts—particularly those employing different tracking technologies or data-processing procedures—remains unclear. Future studies should employ larger and more heterogeneous samples across multiple teams, leagues, and competitive levels to establish normative reference values. In the meantime, practitioners are advised to derive team-specific or individual thresholds where feasible, either through dedicated isolated sprint tests [19] or through systematic training and/or game observations, as applied in the present study.

## 5. Conclusions

This study provides additional empirical evidence, for the first time in the context of ice hockey, that maximal voluntary acceleration capacity is linearly affected by the locomotion speed preceding an acc_effort_ and, therefore, underpins the importance of employing a rel_threshold_ adjusted to the initial skating speed to assess acc_efforts_. Accordingly, we suggest using a relative acceleration threshold to assess acc_efforts_, as it provides a context-sensitive characterization of acc_effort_ magnitude relative to the initial skating speed, which in turn enables high-intensity acc_efforts_ to be identified across a wide range of skating speeds, while avoiding a potential overestimation of submaximal acc_efforts_ initiated from ‘standing’ or ‘very low’ skating speeds. The ability to assess acc_efforts_ initiated at higher skating speeds is particularly relevant for forwards, as it reflects their typical in-game actions and reveals positional differences in acc_efforts_ demands that would otherwise remain undetected. In summary, our observations align with established theoretical frameworks and previous empirical evidence from soccer, suggesting that applying a relative acceleration threshold offers a conceptually more valid approach to assessing acc_efforts_ and is therefore of interest for both applied ice hockey research and training monitoring purposes. Nevertheless, the methodological approach adopted in the present study for detecting players’ discrete acc_efforts_ and for modeling a_max_–v_init capacities_ to derive the relative acceleration threshold should be regarded as exploratory, and further refinement and formal validation in ice hockey remain necessary.

## Figures and Tables

**Table 1:** Descriptive statistics for both computational methods showing the mean number of acc_efforts_ per effective playing minute, paired *t*-test results, and Cohen’s *d* (±95% confidence Interval) overall and across different initial-skating-speed bands.

	fix_threshold_2_	rel_threshold_75%_			
Initial-Skating-Speed Band (m·s^−1^)	acc_efforts_ (Count·min^−1^)	acc_efforts_ (Count·min^−1^)	*t*(21)	*p*	Cohen’s *d* (±95% CI)
All	3.03 ± 0.39	1.65 ± 0.38	−20.10	0.001	4.29 (2.93 to 5.63)
(0–2) very low	2.45 ± 0.32	0.82 ± 0.19	−33.03	0.001	7.04 (4.88 to 9.19)
(2–4) low	0.54 ± 0.12	0.37 ± 0.09	−14.41	0.001	3.07 (2.05 to 4.08)
(4–6) moderate	0.04 ± 0.03	0.33 ± 0.13	12.46	0.001	−2.66 (−3.55 to −1.74)
>6 fast	0.00 ± 0.00	0.13 ± 0.08	7.52	0.001	−1.60 (−2.23 to −0.96)

## Data Availability

The data presented in this study are available on request from the corresponding author. Access is restricted for ethical and privacy reasons, as positional tracking data in conjunction with publicly available video material may enable the re-identification of participants.

## Abbreviations

The following abbreviations are used in this manuscript:

LPS	Local Positioning System
acc_efforts_	Acceleration efforts
rel_threshold_	Relative acceleration threshold
fix_threshold_	Fixed acceleration threshold
a_max_–v_init_	Maximal acceleration–initial velocity
a_distance_	Acceleration effort distance
a_duration_	Acceleration effort duration
v_delta_	Speed increase during the acceleration effort
IIHF	International Ice Hockey Federation
MED	Minimal effort duration
